# Utilization of Simulation to Teach Cardiac Auscultation: A Systematic Review

**DOI:** 10.7759/cureus.41567

**Published:** 2023-07-08

**Authors:** Harrison A Patrizio, Riley Phyu, Bum Kim, Nils V Brolis

**Affiliations:** 1 Department of Clinical Education and Assessment Center, Rowan-Virtua School of Osteopathic Medicine, Stratford, USA; 2 Department of Simulation, Rowan-Virtua School of Osteopathic Medicine, Stratford, USA

**Keywords:** teaching, education, simulation, medical students, cardiac auscultation

## Abstract

This systematic review, following the Preferred Reporting Items for Systematic Reviews and Meta-Analyses (PRISMA) guidelines, evaluates the effectiveness of simulation-based education in teaching cardiac auscultation. A team of researchers conducted a comprehensive, systematic search of the PubMed database from 2010 to 2021, focusing on cardiac auscultation, education, proficiency, and students. After rigorous filtering, a total of 14 articles, primarily involving medical students and residents, met the inclusion criteria. The articles were categorized based on their focus areas: diagnostic accuracy, knowledge acquisition, competency, and learner satisfaction. Findings suggest that the majority of the studies (86% or 12 out of 14) reported positive outcomes of using simulation for teaching cardiac auscultation, demonstrating improvements in the identified focus areas across diverse contexts. The review underscores the need for future research to further standardize simulation teaching practices, aiming to reduce costs, improve usability, and possibly incorporate multiple simulation approaches in a universal educational process. This approach could enhance outcomes across varied fields and learning styles.

## Introduction and background

Since its invention in 1816 by Rene-Theophile-Hyacinthe Laënnec, the stethoscope has emerged as an integral tool in medicine, enabling the diagnosis of numerous cardiac pathologies through the aural investigation of the human body [1]. Early detection of conditions such as cardiac murmurs, often the initial sign of congenital heart disease (CHD), allows for timely referrals and effective management [2]. However, this tool's efficacy is largely contingent on the user's expertise. Inexperience or inadequate training often culminates in incorrect referrals, escalating financial stress, and delaying treatment of time-sensitive diseases. Therefore, the importance of solid training in basic examination skills, particularly in cardiac auscultation, cannot be overstated. Recent studies underscore a worrying trend of dwindling auscultation competency among medical professionals, emphasizing an urgent need for rejuvenation [3-5]. Notably, a study by Mangione S involving 314 internal medicine residents from three countries (United States, Canada and England) revealed diminished cardiac auscultation skills [5], a finding corroborated by Mookherjee S et al.'s review of 14 studies [5]. These findings underscore the imperative for robust, effective educational methodologies in training. Cardiac auscultation is a skill and cannot be learned without practice and feedback; it necessitates considerable practice and hands-on clinical experience [3-5]. While traditional teaching methods have relied on bedside or classroom lectures [6], recent studies have begun to investigate the potential benefits of simulation-based learning in teaching cardiac auscultation. This approach offers a safe learning environment, allowing for trial and error without compromising patient safety. Its efficacy is well-documented in other medical disciplines, such as Emergency Medicine, where it has been shown to significantly enhance team performance, clinical knowledge, procedural skills, and communication [7]. Despite ongoing research into simulation-based education for cardiac auscultation, comprehensive insights into the resultant learning outcomes remain limited. A review article by Issenberg SB et al. provided similar insight; however, this review consisted of articles between 1969 and 2003 and therefore lacks consideration of the most current research [8]. This paper presents a systematic review adhering to the Preferred Reporting Items for Systematic Reviews and Meta-analyses (PRISMA) 2009 guidelines, appraising and synthesizing existing research to explore the potential benefits of simulation-based training in cardiac auscultation education [9].

## Review

Methods

Search Process

**Figure 1:**
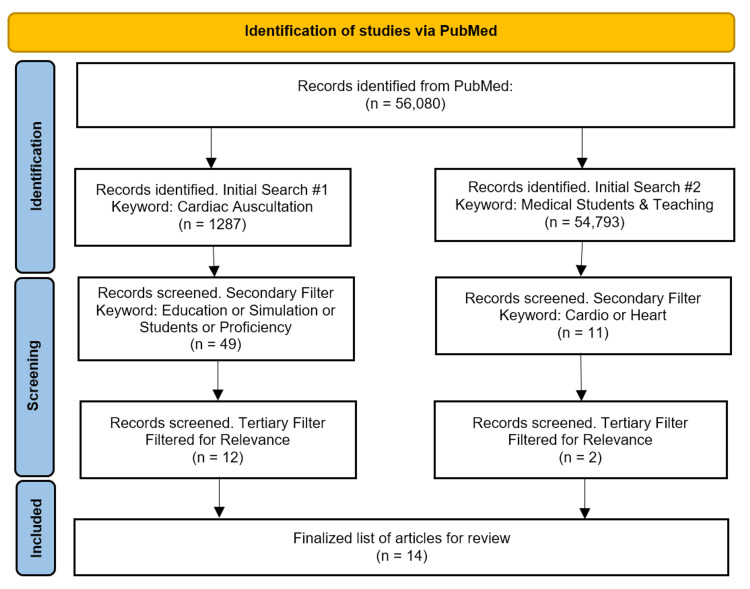
PRISMA article selection flowchart.

A team of three researchers conducted a comprehensive and systematic search of the literature using the PubMed database, focusing on the effectiveness of implementing simulation in medical education. The PRISMA guidelines were used to structure the search [10]. Articles were included based on the following criteria. We included all primary research types focusing on cardiac auscultation, which would allow us to properly assess the results and conclusions of each paper ourselves and maximize the article pool. We also did not discriminate between the different simulation types to increase the included articles. Simulation education was defined as utilizing physically interactive models that simulate real life to teach cardiac auscultation. All types of medical professionals were also included. Articles were excluded based on the following criteria: non-primary research, non-English articles, only qualitative data, irrelevant use of simulation, and published over 10 years ago. These exclusion criteria allowed us to ensure proper understanding, analysis, and consistency across all reviewers. Two initial searches were performed to broaden the article pool. Initial search #1 used the keyword "Cardiac Auscultation," resulting in 1287 articles. A secondary filter was then applied to narrow the results to only education results using ("Education" OR "Simulation" OR "Students" OR "Proficiency"). This reduced the pool to 49 articles. Initial search #2 using the keywords ("Medical students" AND "Teaching") resulted in an additional 54,793 articles. The secondary filter consisted of ("Cardio" OR "Heart") to provide the proper cardiac-related context reducing the pool to 11 articles. The tertiary filter for both queries consisted of two researchers independently reviewing article abstracts for our outlined criteria. If either reviewer suggested inclusion, the article was kept. After all the articles were collected, a full-text review was initiated, and all three researchers' disagreements on inclusion were resolved with a group vote to determine inclusion based on the outlined criteria. This process left a total of 14 articles for inclusion (Figure 1).

Data Extraction and Synthesis

All studies were meticulously analyzed for intervention patterns and summarized in Table 1. The summary table included pertinent and relevant information for analysis, such as first author, publication year, study design, sample size, intervention summary, and assessment type. Assessment types from each study were categorized into one or more of the following outcomes: diagnostic accuracy, knowledge acquisition, competency, and learner satisfaction (which included self-confidence and anxiety scores). We also recorded each study's country of origin and funding source to provide a better context for the literature batch. Due to the variety of outcomes measured, a formal meta-analysis was not performed. Instead, we categorized each study based on the presence of positive or no difference in outcomes. The label of positive outcomes was assigned to articles presenting substantial evidence that the simulation intervention outperformed the control group. On the other hand, no difference in outcomes was assigned to articles where the simulation intervention did not demonstrate superiority over the control group. κ calculation was performed to measure agreement across reviewers for study inclusion [11]. We then analyzed the data using two separate quality metrics to add more validity to any conclusions our review provided. The Best Evidence in Medical Education (BEME) was used to provide the level of evidence quality in each article (Table 2) [12]. Kirkpatrick's levels for outcomes were calculated to more easily convey the impact each educational technique had on the participants (Table 2) [12]. Intraclass correlation was performed for agreement between reviewers in Kirkpatrick's levels for outcomes [11]. All data statistics were performed using IBM SPSS.

**Table 1:** Invention descriptions and outcome measures for included studies. *= Not Significant, †= Self Confidence, ††= Anxiety Score. PE: Pulmonary embolism; RV: Right ventricle; MI: Myocardial ischemia; ASD: Atrial septal defect; VSD: Ventricular septal defect; COA: Coarctation of aorta; ToF: Tetralogy of Fallot.

Author, year	Sample size	Intervention Description	Diagnostic Accuracy (P-value)	Knowledge Acquisition (P-value)	Competency (P-value)	Learner Satisfaction (P-value)
Bernardi S et al. (2019) [13]	n= 109	1 hour training with Kyoto Kagaku (a patient simulator)	0.02			
Binka EK et al. (2016) [14]	n = 108	45 minutes training module on cardiac auscultation	<0.001			
Butter J et al. (2010) [15]	n = 108	Computer based interactive self study tutorial which featured didactic instruction, deliberate practice and self assessment.	<0.001			
Fraser K et al. (2011) [16]	n = 86	Simulation sessions with clinical scenarios of acute onset chest pain (PE with RV strain but no murmur, symptomatic Aortic stenosis, or MI causing mitral regurgitation)	0.01			
Friederichs H et al. (2014) [17]	n = 142	Hybrid model included standard patients electronically fitted to produce pathological heart sounds.				<0.001
Karsenty C et al. (2021) [18]	n = 347	Physical 3D models of the heart with congenital heart defects (ASD, VSD, COA, ToF)		<0.001	<0.001	<0.001
Kern DH et al. (2011) [19]	n = 405	Received both standardized patient and cardiac simulation.			0.004	
Martínez G et al. (2012) [20]	n = 32	Training program in cardiac auscultation using Student Auscultation Manikin (SAM).	<0.001			
Perlini S et al. (2012) [21]	n = 627	10 hour teaching session with Harvey	<0.001			
Sverdrup Ø et al. (2010) [22]	n = 49	4 hours of auscultation training with CardioSim Auscultation System.	0.689 *			
Watsjold B et al. (2021) [23]	n = 135	Cardiac sounds were presented in combination with phonocardiogram tracings.	0.035			
Vural Doğru B and Zengin Aydın L (2019) [24]	n = 72	Cardiac auscultation training with a high fidelity stimulator.		<0.001	<0.001	<0.001 ††
Goldsworthy S et al. (2010) [25]	n = 127	3 auscultation learning sessions that were each 2 hours in length (cardiac, pulmonary and mixed sounds).	0.019			
Loke YH et al. (2017) [26]	n = 35	Physical 3D models printed from 3D cardiac imaging data sets (cardiac MR, CT, 3D echocardiogram).		>0.05 *		0.03 0.39 †*

**Table 2:** Overview of the Kirkpatrick levels and Best Evidence of Medical Education (BEME) appraisal scales. Adapted from [12]. Definitions used to determine the placement of the Kirkpatrick levels and Best Evidence of Medical Education Appraisal Scales.

Kirkpatrick levels	
Level 1	Participation - Covers learners' views on the learning experience, its organization, presentation, content, teaching methods, and aspects of the instructional organization, materials, and quality of instruction.
Level 2a	Modification of Attitudes/Perceptions - Outcomes relate to changes in the reciprocal attitudes or perceptions between participant groups towards the intervention/simulation.
Level 2b	Modification of Knowledge/Skills - For knowledge, this relates to the acquisition of concepts, procedures, and principles. For skills, this relates to the acquisition of thinking/problem-solving, psychomotor, and social skills.
Level 3	Behavioral Change - Documents the transfer of learning to the workplace or the willingness of learners to apply new knowledge and skills.
Level 4a	Change in Organizational Practice - Wider changes in the organization or delivery of care, attributable to an educational program.
Level 4b	Benefits to Patients/Clients - Any improvement in the health and well-being of patients/clients as a direct result of an educational program.
BEME Appraisal Scale	
1	No clear conclusions can be drawn; not significant.
2	Results ambiguous, but there appears to be a trend.
3	Conclusions can probably be based on the results.
4	Results are clear and very likely to be true.
5	Results are unequivocal.

Results

After individual review and resolution of differences, 14 studies were included [13-26]. The agreement between reviewers on inclusion was exceptional (κ = 0.902, 95% CI = 0.77-1.04, P < 0.001). The articles spanned from 2010 to 2021. All articles were written in English and available for full-text review.

Learners and Content

A total of 2432 learners were included in these studies spanning from 2010 to 2021, with 1507 receiving simulation-based teaching and 925 in control groups who did not receive simulation-based training. The learners represented the four populations: medical students (n=11) [13-23], nursing students (n=2) [23,24], and pediatric residents (n=1) (Table 3) [26]. The simulation technology used in the studies varied widely. The simulations included Kyoto Kagaku [13], High-Fidelity Simulation [24], Harvey [21], Student Auscultation Mannequin (SAM) [20], and CardioSim Auscultation System [22]. Most studies (36%, n=5) were conducted in the US [14,15,19,23,26], 14% (n= 2) were conducted in Canada [16,25], 14% (n= 2) were conducted in Italy [13,21], and 7% (n= 1) each from Turkey [24], Germany [17], France [18], Chile [20], and Norway [22] (Table 3).

**Table 3:** Population and study locations (n=14).

Study Populations (n)	
Medical Students	11
Nursing Students	2
Pediatric Residents	1
Study Locations (n)	
USA	5
Canada	2
Italy	2
Turkey	1
Germany	1
France	1
Chile	1
Norway	1

Rigor and Quality of Studies

Most of the articles included in the review were randomized controlled trials (n=7) [17,20,22-24,26] and prospective studies (n=5) [13,14,16,18,21]. In contrast, quasi-experimental studies [19] and non-randomized studies [15] made up the minority contributing to one study each (Table 4). When analyzed using the BEME appraisal system [12], 43% (n= 6) of the studies received a rating of 4 [15,17-19,21,24], 43% (n=6) of the studies received a rating of 3 [13,15,16,20,23,25], 14% (n=2) of the studies received a rating of 2 [22,26], none of the studies received a rating of one. Ten studies (71%) reported no sources of funding [13,14,16-19,21,23,26] (Table 4). The remaining studies received internal funding (29%, n=4) [15,20,23,25] from their respective programs (Table 4).

**Table 4:** Quality rankings, study types, Kirkpatrick levels, and BEME levels (n=14). BEME: Best Evidence of Medical Education.

Study Type (n)	
Prospective	5
Randomized Controlled	7
Non-Randomized Controlled	1
Quasi-experimental	1
Type of Funding	
None	10
Internal	4
BEME Level (n)	
2	2
3	6
4	6
Kirkpatrick Level (n)	
2a	3
2b	9
2a & 2b	2

Outcomes

Of the 14 included articles, 12 (86%) showed the benefits of simulation education in cardiac auscultation [13-21]. One study by Loke YH et al. showed no difference in outcome. It was a randomized controlled study of 35 pediatric residents assessing knowledge acquisition and learning satisfaction using 2D and 3D models of a Tetralogy of Fallot pathology [26]. Another study by Sverdrup Ø et al. assessing 49 third-year medical students also showed no difference in outcome. No statistical difference was found between groups in diagnostic accuracy using the CardioSim Auscultation System [22]. All studies (n=14) were identified to be Kirkpatrick level 2a or 2b (modification of perception/attitudes/knowledge/skills) [12]. The intraclass correlation coefficient for Kirkpatrick was 0.83 (95% CI = 0.524-0.938, P < 0.001) (Table 4).

Discussion

This literature review showed that the utilization of simulation in cardiac auscultation is being researched globally, with most research focusing on the USA. The analysis of the BEME grading scale yielded positive results, with 86% of studies ranked 3 (n=6) or 4 (n=6), suggesting that the study conclusions can be looked at with increased validity. Kirkpatrick level rankings varied to include learner satisfaction (Kirkpatrick 2a) and improvements in knowledge and skills (Kirkpatrick 2b). The included articles are also spread across a wide variety of countries, which may improve the generalizability of the results. The distribution of participants is logical, given the importance of cardiac auscultation in the medical field. Most of the studies concentrated on medical students (n=11), reflecting the pivotal role of auscultation in cardiology and the importance of equipping future physicians with this critical diagnostic skill. Notably, studies involving nursing students (n=2) and pediatric residents (n=1) illustrate the relevance of this skill in broader clinical settings. For example, early detection of cardiac anomalies in children by pediatricians can result in improved patient outcomes. The lack of diversity in the populations studied could be due to the relative importance of auscultation skills in different healthcare disciplines. Paramedics, while they could benefit from improved proficiency in cardiac auscultation, may prioritize more emergency-oriented skills due to the urgent nature of their role [27]. Similarly, the high cost of healthcare simulators, which can exceed $60,000, might deter their adoption in fields where auscultation may not be as critical or other diagnostic tools like ultrasound are more commonly used [28]. The studies also employed various methods of cardiac auscultation. For example, the High Fidelity Simulation used by Vural Doğru B and Zengin Aydın L demonstrated improvements in knowledge, diagnostic skill, and performance anxiety among the nursing students, suggesting the potential for anxiety reduction as an added benefit of simulation training [24]. On the other hand, the study by Goldsworthy S et al., which included additional virtual auditory learning sessions, reported significant improvements in recognizing normal versus abnormal heart sounds [25]. The different training modalities shown in these studies reflect the value of diversifying simulation methods to cater to the learning needs of different healthcare providers. However, not all studies using these interventions reported universally positive outcomes. For instance, Sverdrup Ø et al.' study, which assessed 49 third-year medical students, reported no significant difference in diagnostic accuracy between the group that received additional four hours of auscultation training with conventional bedside training and the group that received additional four hours of auscultation training with CardioSim Auscultation System [22]. This outcome might be attributed to the study's small sample size of 49 third-year medical students and the fact that both groups experienced a similar training style, one via simulation and the other in a real clinical setting. Since simulation aims to mimic real-life scenarios, this overlapping approach could account for the lack of observed difference.

Similarly, Loke YH et al.'s study, involving 35 pediatric residents, reported mixed results [26]. Despite significant improvements in learner satisfaction, there were no significant improvements in the knowledge or confidence levels of the residents. This might be due to the study's specific focus on the Tetralogy of Fallot, a common congenital heart condition. Given the residents' familiarity with this condition, the simulation training may not have significantly impacted their existing knowledge or confidence levels. However, the significant improvement in learner satisfaction could be attributed to the engaging and interactive nature of the simulation. Despite these limitations, the remaining studies involving medical students consistently showed positive outcomes when using various simulation techniques for teaching cardiac auscultation compared to traditional teaching methods. This suggests that a variety of simulation methods could be a powerful tool in teaching future healthcare professionals to accurately detect and diagnose heart conditions. The high BEME ranking, a metric used to evaluate the quality of education studies, further validates the positive outcomes observed in these studies. However, reaching higher Kirkpatrick levels, a model that measures the effectiveness of training is more challenging. Higher levels in this model denote not just the acquisition of skills but also their application in the workplace and their direct benefits to patients. Assessing these higher levels can be complex due to various potential confounding factors. For instance, it would be challenging to definitively attribute improved patient outcomes to simulation training, as various other factors such as patient health, other treatments, and real-world experience could also play a role. Despite these complexities, the overall high BEME rankings of these studies suggest that the conclusions made about the effectiveness of simulation in teaching cardiac auscultation are reliable. The strong results from these studies bolster the argument for incorporating simulation techniques into medical education. In conclusion, the review underscores the continued effectiveness of integrating simulation components into cardiac auscultation education in recent years. However, barriers like high initial costs, funding allocation issues, and lack of standardized simulation teaching tools could inhibit broader implementation. Future research should focus on finding solutions to these challenges, such as developing affordable and user-friendly tools and programs. Additionally, a more synergistic education approach, rather than focusing solely on High-Fidelity Simulation or 3D modeling, might provide deeper insights into how healthcare providers can effectively master the art of cardiac auscultation.

Limitations

Despite the substantial insights offered by this review, it comes with its own limitations that need to be addressed. One of the principal limitations lies in the methodological approach. This review did not utilize standard techniques employed in a meta-analysis, leaving some biases within the reviewed studies unaccounted for. As such, the impact of these potential biases on the overall conclusions of the review remains uncertain. Another limitation of our study stems from the wide range of learner groups in assessing simulation-based teaching effectiveness. The variety in experience levels, from early preclinical medical students to qualified clinicians, introduces substantial variability in our results. The effectiveness of simulation-based learning likely differs based on the learner's prior clinical experience, comfort with technology, and individual learning styles. Thus, both novices and seasoned practitioners might derive differing benefits from this pedagogical approach. As pointed out in one of the reviews, this broad inclusion of learner groups can cloud the overall conclusions. A more stratified approach targeting specific learner cohorts in future studies could yield more definitive and applicable results. This could help educators to better tailor simulation-based teaching methods to suit specific learner needs and ultimately enhance the teaching of cardiac auscultation. Finally, another limitation centers around the study population. The studies considered in this review predominantly concentrate on medical students, with a lesser emphasis on other vital practitioners such as nurses or pediatric residents. Therefore, the potential benefits and applicability of simulation training for cardiac auscultation among these groups might be underrepresented. This underscores the need for further research that includes a more diverse population of healthcare providers, encompassing a wider range of disciplines and experience levels. The cost factor associated with simulation technology is another potential limitation. The high initial investment and maintenance costs might deter certain institutions from incorporating simulation-based learning. As a result, the findings of this review might not be generalizable to institutions with limited financial resources. Lastly, the nature of the review might have led to publication bias, as studies with negative outcomes may not have been published or included. This bias could potentially overstate the effectiveness of simulation-based cardiac auscultation training. Accounting for these limitations, the findings of this review provide a compelling case for integrating simulation-based learning in cardiac auscultation education. However, future research should aim to address these limitations to provide a more comprehensive understanding of the benefits and potential drawbacks of simulation in cardiac auscultation training.

## Conclusions

Using simulation to teach cardiac auscultation improves diagnostic accuracy, knowledge acquisition, learner satisfaction, user anxiety and confidence levels, and overall competence across a diverse set of locations and populations. Future research should address the creation of more standard approaches to simulation to reduce cost and improve usability. Additionally, the inclusion of multiple simulation methods into a single universal education process may yield improved outcomes across many fields and learning styles.

## References

[1] Roguin A (2006). Rene Theophile Hyacinthe Laënnec (1781-1826): the man behind the stethoscope. Clin Med Res.

[2] Montinari MR, Minelli S (2019). The first 200 years of cardiac auscultation and future perspectives. J Multidiscip Healthc.

[3] Vukanovic-Criley JM, Criley S, Warde CM (2006). Competency in cardiac examination skills in medical students, trainees, physicians, and faculty: a multicenter study. Arch Intern Med.

[4] Mookherjee S, Pheatt L, Ranji SR, Chou CL (2013). Physical examination education in graduate medical education--a systematic review of the literature. J Gen Intern Med.

[5] Mangione S (2001). Cardiac auscultatory skills of physicians-in-training: a comparison of three English-speaking countries. Am J Med.

[6] Asghar O, Alam U, Khan S, Hayat S, Malik RA (2010). Cardiac auscultation: the past, present and future. British J Cardiol.

[7] McFetrich J (2006). A structured literature review on the use of high fidelity patient simulators for teaching in emergency medicine. Emerg Med J.

[8] Issenberg SB, McGaghie WC, Petrusa ER, Lee Gordon D, Scalese RJ (2005). Features and uses of high-fidelity medical simulations that lead to effective learning: a BEME systematic review. Med Teach.

[9] Moher D, Liberati A, Tetzlaff J, Altman DG (2009). Preferred reporting items for systematic reviews and meta-analyses: the PRISMA statement. Ann Intern Med.

[10] Liberati A, Altman DG, Tetzlaff J (2009). The PRISMA statement for reporting systematic reviews and meta-analyses of studies that evaluate healthcare interventions: explanation and elaboration. BMJ.

[11] Landis JR, Koch GG (1977). The measurement of observer agreement for categorical data. Biometrics.

[12] Yardley S, Dornan T (2012). Kirkpatrick's levels and education 'evidence'. Med Educ.

[13] Bernardi S, Giudici F, Leone MF, Zuolo G, Furlotti S, Carretta R, Fabris B (2019). A prospective study on the efficacy of patient simulation in heart and lung auscultation. BMC Med Educ.

[14] Binka EK, Lewin LO, Gaskin PR (2016). Small steps in impacting clinical auscultation of medical students. Glob Pediatr Health.

[15] Butter J, McGaghie WC, Cohen ER, Kaye M, Wayne DB (2010). Simulation-based mastery learning improves cardiac auscultation skills in medical students. J Gen Intern Med.

[16] Fraser K, Wright B, Girard L, Tworek J, Paget M, Welikovich L, McLaughlin K (2011). Simulation training improves diagnostic performance on a real patient with similar clinical findings. Chest.

[17] Friederichs H, Weissenstein A, Ligges S, Möller D, Becker JC, Marschall B (2014). Combining simulated patients and simulators: pilot study of hybrid simulation in teaching cardiac auscultation. Adv Physiol Educ.

[18] Karsenty C, Guitarte A, Dulac Y (2021). The usefulness of 3D printed heart models for medical student education in congenital heart disease. BMC Med Educ.

[19] Kern DH, Mainous AG 3rd, Carey M, Beddingfield A (2011). Simulation-based teaching to improve cardiovascular exam skills performance among third-year medical students. Teach Learn Med.

[20] Martínez G, Guarda E, Baeza R, Garayar B, Chamorro G, Casanegra P (2012). A heart sound simulator as an effective aid in teaching cardiac auscultation to medical students and internal medicine residents. Rev Esp Cardiol.

[21] Perlini S, Salinaro F, Santalucia P, Musca F (2014). Simulation-guided cardiac auscultation improves medical students' clinical skills: the Pavia pilot experience. Intern Emerg Med.

[22] Sverdrup Ø, Jensen T, Solheim S, Gjesdal K (2010). Training auscultatory skills: computer simulated heart sounds or additional bedside training? A randomized trial on third-year medical students. BMC Med Educ.

[23] Watsjold B, Ilgen J, Monteiro S, Sibbald M, Goldberger ZD, Thompson WR, Norman G (2021). Do you hear what you see? Utilizing phonocardiography to enhance proficiency in cardiac auscultation. Perspect Med Educ.

[24] Vural Doğru B, Zengin Aydın L (2020). The effects of training with simulation on knowledge, skill and anxiety levels of the nursing students in terms of cardiac auscultation: a randomized controlled study. Nurse Educ Today.

[25] Goldsworthy S, Gomes P, Coimbra M, Patterson JD, Langille J, Perez G, Fasken L (2021). Do basic auscultation skills need to be resuscitated? A new strategy for improving competency among nursing students. Nurse Educ Today.

[26] Loke YH, Harahsheh AS, Krieger A, Olivieri LJ (2017). Usage of 3D models of tetralogy of Fallot for medical education: impact on learning congenital heart disease. BMC Med Educ.

[27] Jurkovich GJ, Campbell D, Padrta J, Luterman A (1987). Paramedic perception of elapsed field time. J Trauma.

[28] Damp J, Anthony R, Davidson MA, Mendes L (2013). Effects of transesophageal echocardiography simulator training on learning and performance in cardiovascular medicine fellows. J Am Soc Echocardiogr.

